# Motivations for testing and linkage to HIV services following HIV self-testing among fishermen in Kenya: a qualitative analysis

**DOI:** 10.1080/09540121.2026.2676207

**Published:** 2026-06-09

**Authors:** Holly Nishimura, Kawango Agot, Sarah A. Gutin, Phoebe Olugo, Jayne Lewis-Kulzer, Zachary Kwena, Marguerite Thorp, Benard Ayieko, Harsha Thirumurthy, Carol S. Camlin

**Affiliations:** a Department of Medicine, Division of Prevention Science, University of California San Francisco, San Francisco, CA, USA;; b Impact Research and Development Organization, Kisumu, Kenya;; c Department of Community Health Systems, School of Nursing, University of California San Francisco, San Francisco, CA, USA;; d Department of Obstetrics, Gynecology and Reproductive Sciences, University of California San Francisco, San Francisco, CA, USA;; e Centre for Microbiology Research, Kenya Medical Research Institute, Kisumu, Kenya;; f Division of Infectious Diseases, University of California, Los Angeles, Los Angeles, CA, USA;; g Center for Health Incentives and Behavioral Economics, University of Pennsylvania, Philadelphia, PA, USA

**Keywords:** Kenya, fishermen, men, HIV self-testing, linkage to care, SDG 3: Good health and well-being, SDG 10: Reduced inequalities

## Abstract

HIV self-testing (HIVST) is a promising strategy to expand testing access for hard-to-reach populations such as Kenyan fishermen. We examined motivations and barriers to HIVST use and linkage to HIV prevention and treatment services (including PrEP or ART), within a social network-based intervention (NCT04772469). We conducted interviews (*N* = 64) and focus group discussions (*N* = 4) with fishermen in Siaya County, including 30 “promoters” who distributed HIVST kits and transportation vouchers within their social networks. Transcribed interviews were coded using framework analysis with a mixed inductive–deductive codebook. Convenience, privacy and avoidance of stigma motivated HIVST uptake. Men perceiving themselves at high HIV risk were especially motivated to test, while some preferred clinic-based testing for counseling and perceived accuracy. Promoter training increased personal and peer testing motivation. Confirmatory testing was facilitated by perceived HIV risk, PrEP interest, spousal encouragement and transport vouchers offsetting travel costs. Barriers included distance to clinics, competing work priorities, mobility and the perception that confirmatory testing was unnecessary after a negative result. Gender norms prioritizing work over healthcare further impeded linkage. HIVST is a valuable tool for increasing testing among highly mobile men and its impact can be strengthened through social network-based delivery.

## Introduction

Men in fishing communities along Lake Victoria, Kenya, where HIV prevalence reaches up to 31%, remain disproportionately affected by HIV and are persistently underserved by conventional testing, prevention, and treatment services (Kwena et al., 2013; Lewis-Kulzer et al., 2025; Olugo et al., 2025; Silva, 2013). Fishermen face unique social and structural barriers, including occupational mobility, HIV stigma and limited clinic use, contributing to low rates of HIV status awareness, prevention uptake and engagement in HIV care (Olugo et al., 2025; Sileo, Reed, et al., 2019a; Sileo, Wanyenze, et al., 2019b; Sileo et al., 2021; Tumwine et al., 2020). In Siaya County, only 61% of adult men living with HIV were virally suppressed in 2019, compared to 74% of women, indicating a gender gap in treatment access and adherence (Kenya Ministry of Health, 2020). Prior qualitative research has documented low awareness and misconceptions about HIV prevention options such as pre-exposure prophylaxis (PrEP) among fishermen, suggesting limited engagement with prevention services even when testing occurs (Olugo et al., 2025). These service gaps underscore the need for targeted, scalable strategies to enhance uptake of services across the HIV care continuum.

HIV self-testing (HIVST) has the potential to facilitate progress toward the UNAIDS 95–95-95 targets by offering a convenient and private testing option for populations that are otherwise difficult to reach (Indravudh et al., 2021; Johnson et al., 2014). HIVST has shown promise as a tool to increase testing coverage among men who are less likely to access facility-based services (d’Elbee et al., 2018; Johnson et al., 2017). However, increased testing does not necessarily translate into engagement with HIV prevention or treatment services. A 2023 systematic review of HIVST implementation in sub-Saharan Africa identified inconsistent patterns of post-test linkage, highlighting persistent behavioral and structural barriers (Hlongwa et al., 2023). A more recent global systematic review and meta-analysis similarly found that while HIVST increases uptake of HIV testing, the effects on linkage to confirmatory testing, prevention and treatment services vary widely, with multi-component interventions with those combining HIVST with financial incentives, peer or lay provider follow-up and facilitated referral demonstrating the most promise (Adeagbo et al., 2024).

Barriers to linkage most frequently cited for men in sub-Saharan Africa encompass travel-related expenses (Chipungu et al., 2017; Choko et al., 2019; Korte et al., 2020), lengthy clinic wait times (Conserve et al., 2018; Korte et al., 2020), experiences of stigma and discrimination and worries about maintaining privacy (Conserve et al., 2018; Rujumba et al., 2021). Incentives (Choko et al., 2019) and social support (Matovu et al., 2020) have emerged as potential facilitators. In fishing communities, these barriers are intensified by high mobility, economic precarity and gendered norms that may prioritize income generation and breadwinning over preventive healthcare, unless illness threatens the ability to work (Sileo, Reed, et al., 2019a). Despite growing recognition that linkage is a critical bottleneck following HIVST, few studies have examined how men in highly mobile fishing communities navigate decisions about confirmatory testing, PrEP initiation, or ART engagement after self-testing.

The “Owete” trial (NCT04772469) leveraged men’s social networks to improve HIVST and linkage outcomes among fishermen in western Kenya (Sheira et al., 2022). The trial identified socially network-central men (“promoters”) within fishing communities and trained them to distribute HIVST kits and support linkage within their networks. While quantitative analyses from the Owete trial demonstrated improvements in HIV testing and clinic attendance (Camlin et al., 2025), they provide limited insight into the mechanisms shaping men’s decisions about linkage following HIVST. A prior qualitative analysis using baseline qualitative data from the Owete trial examined participants’ knowledge of and attitudes toward HIVST before its introduction in the community, focusing on anticipated use and acceptability and substantial willingness to try HIVST (Lewis-Kulzer et al., 2025). Less is known about how men experienced HIVST in practice and how anticipated benefits aligned or diverged from lived experiences once HIVST was available.

This qualitative study addresses these gaps by drawing on in-depth interviews and focus group discussions with fishermen in Siaya County, Kenya, conducted three months after HIVST distribution in the Owete trial. We examine: (1) How do fishermen describe their motivations for and experiences with HIVST? and (2) What factors facilitate or impede linkage to confirmatory testing and HIV prevention services following HIVST? By focusing on fishermen’s experiences of HIVST and linkage, this study aims to inform the design of behavioral and social network-based strategies to strengthen engagement across the HIV prevention continuum among mobile and hard-to-reach populations.

## Methods

### Owete trial

Qualitative data are from the study *Self-Test Strategies and Linkage Incentives to Improve ART and PrEP Uptake in Men* [NCT 04772469], or *Owete*, a cluster randomized controlled trial designed to evaluate an HIV testing and linkage-to-care intervention among highly mobile fishermen in western Kenya (Sheira et al., 2022). The study was conducted in three fishing communities in Siaya County, located along the shores of Lake Victoria. HIV prevalence in Siaya County was estimated to be 15.3% in 2018, compared to the national prevalence of 4.9% (Kenya Ministry of Health, 2022). The trial employed an HIV status–neutral approach, identifying highly socially connected men (“promoters”) within close-knit male networks. These promoters, along with their network clusters, were randomized to either the intervention or control arm. Promoters in both arms received basic HIV education. Those in the intervention arm received additional training to distribute oral-fluid HIVST kits, provide transport vouchers, and share information on linkage to antiretroviral therapy (ART) and pre-exposure prophylaxis (PrEP) to other men in their networks. Participants in the intervention arm also received 500 Ksh (~$3.50 USD) transport vouchers to reduce financial barriers to clinic access. Control arm promoters distributed vouchers that could be redeemed for free HIV testing at local clinics. Intervention activities began immediately after promoter training.

### Sampling and recruitment

Fishermen were purposively sampled from the full cohort enrolled in the Owete trial, drawing from men who had completed baseline enrollment. Sampling was designed to ensure variation across key characteristics relevant to the intervention, including study arm (intervention vs. control), study role (promoter vs. cluster member), age group (<35 vs. ≥35 years) and fishing community. Inclusion criteria were enrollment in the Owete trial, active engagement in fishing activities within the study communities and the ability to provide informed consent. Exclusion criteria included absence from the community during the interview period or inability to provide informed consent. At the three-month follow-up, 64 in-depth interviews (IDIs) were completed, with one eligible participant unreachable due to mobility. Sample size sufficiency was assessed iteratively during analysis, with the research team monitoring the emergence of new themes across participant roles, study arms, and communities. By the later stages of coding, interviews yielded recurring patterns with limited new conceptual insights related to HIVST and linkage, indicating thematic redundancy and supporting the adequacy of the final sample for addressing the study aims (Saunders et al., 2018).

### Data collection

Qualitative data collection occurred within each beach community at baseline, 3-month and 6-month follow-up. Semi-structured interview guides included open-ended questions and probes focused on individual experiences with HIVST, confirmatory testing and engagement in HIV prevention or treatment services, including ART or PrEP and promoters’ roles within social networks (if applicable). English interview guides were translated into Dholuo and Kiswahili and back-translated to ensure accuracy. FGDs facilitated group discussion of shared norms and experiences related to HIV testing and care. Interviews and FGDs were conducted in private rooms within the communities to ensure privacy and confidentiality. IDIs were approximately 60 minutes and FGDs were 90–120 minutes. All sessions were audio-recorded. Field notes were taken during and immediately after IDIs and FGDs to document contextual observations and non-verbal cues. Audio recordings were translated and transcribed into English, and all transcripts were reviewed for transcription and translation quality. Written informed consent was obtained from all participants prior to data collection, and confidentiality was maintained through anonymization and secure storage of audio files and transcripts on password-protected devices accessible only to the study team. Each participant received 500 Kenyan shillings (approximately USD $3.50) for their participation.

### Data analysis

Data analysis was restricted to 3-month follow-up interviews, which corresponds to the period in the trial shortly after HIVST kits were distributed by promoters, and confirmatory testing and linkage would have occurred. The analysis is grounded in a pragmatic qualitative approach that emphasizes participants’ experiences of HIVST and linkage within their social and structural contexts and is oriented toward generating applied insights relevant to intervention design and implementation. The research team used deductive and inductive approaches to develop a codebook, which included identifying a priori codes from interview guides as well as from a sample of initial transcripts. The codebook was then refined after further data review. Six coders (a senior Kenyan social scientist, a Kenyan qualitative research coordinator and four US-based qualitative researchers) separately coded an initial sample of transcripts and then worked together to agree on code application. Discrepancies in code application among the research team were discussed to reach a consensus. After code consensus was reached, each coder individually coded a set of transcripts. Code definitions and any issues with code application were discussed during regular weekly meetings, and codes were edited as needed through team agreement. Dedoose software was used to facilitate data management and coding (SocioCultural Research Consultants, 2023). Further analysis was conducted using a framework analysis approach (Gale et al., 2013), which allowed the researchers to categorize and analyze excerpts related to motivations and barriers to HIVST uptake and linkage to confirmatory testing and HIV services.

### Research team and reflexivity

Data were collected by four Kenyan qualitative researchers with prior experience conducting HIV and sexual health research in fishing communities along Lake Victoria. Interviewers were fluent in Dholuo and Kiswahili and shared cultural and community familiarity with participants, which facilitated rapport and open discussion of sensitive topics. All interviewers received training on qualitative interviewing techniques and research ethics prior to data collection. The broader analytic team included Kenyan and U.S.-based researchers with training in qualitative methods, HIV prevention and social science. Ongoing team discussions during coding and analysis were used to surface assumptions, examine alternative interpretations and promote analytic rigor.

### Ethical approval

This study was approved by the Kenya Medical Research Institute-Scientific Ethics Review Unit (KEMRI-SERU, 677) and the University of California, San Francisco Human Research Protection Program (UCSF-HRPP, 19–2820p. 5). All participants provided written informed consent.

## Results

### Characteristics of study sample

Participants (*N* = 64) were predominantly married (85.9%), with a median age of 34 years (IQR: 29.0–39.1) (Table 1). The majority had primary education or less (70.3%), and nearly all relied on fishing as their primary income source (89.1%). Over half reported no condom use with any partner in the past six months (57.1%), and 38.7% reported concurrent sexual partnerships. At the three-month follow-up, nearly all participants had been tested for HIV (93.8%).

The findings presented below draw on participants’ narratives to explore motivations and barriers to HIVST and linkage to HIV services, with a focus on how fishermen navigated decisions about confirmatory testing and service engagement. We organize the results into two sections aligned with our two research questions: (1) *What motivates men to use HIVST?* and (2) *What factors motivate or impede linkage to confirmatory testing and HIV prevention services following HIVST?* Across themes, participants’ accounts reflected the interaction of individual risk perception, peer influence and structural constraints, including mobility and work demands. Selected quotes are used to illustrate common themes and variation in men’s experiences, with attention to both drivers of engagement and persistent obstacles to care. Quotations are identified by study arm (intervention or control), role (promoter or cluster member) and linkage outcome. Identifiers included with quotes denote the participant’s age, study arm (intervention or control), role in the study (promoter or cluster member) and whether they successfully linked to HIV testing and/or services at a facility (linked to facility or unlinked to facility).

## Motivations for HIVST

### Flexibility and mobility in testing decisions

Men described HIVST as fitting readily into their lives, shaped by irregular work schedules and frequent movement between fishing sites. For those whose work often took them away from clinics or extended beyond clinic hours, HIVST offered a way to test without interrupting income-generating activities. One intervention-arm promoter emphasized the importance of being able to test while mobile:

Because the self-test kit gives me easier time, us fishermen we are people who are always on the move and at times you are far from the hospital but if you have the kit then it is easier for you to test yourself and to know your status. […] you can test yourself anytime and anywhere. It makes it easier for you to know what is happening in your body.

(Intervention arm, promoter, linked to facility)

These accounts were echoed in FGDs, where men collectively described the challenge of aligning clinic-based testing with fishing schedules, reinforcing the importance of flexible testing options for highly mobile fishermen.

### Privacy, stigma and control over disclosure

Beyond convenience, men emphasized privacy as a central motivation for HIVST. Fear of being recognized at clinics, having their name recorded and the potential for someone from their village to find out their HIV status shaped preferences for self-testing. One promoter described how anonymity made HIVST preferable:

Self-testing is better for me than going to the hospital. You don’t use my name, you use a number. But at the hospital they are going to use my name. […] Maybe the person who is going to conduct the test is someone who knows me.–Intervention arm, promoter, linked to facility.

(Intervention arm, promoter, linked to facility)

While HIVST was valued for the privacy it afforded, some men still preferred the structure and support of clinic-based testing. A few participants reported greater trust in results obtained from healthcare providers and appreciated the emotional reassurance provided through pre- and post-test counseling. As one man described, being counseled ahead of time gave him “some confidence before I go for testing” (Intervention arm, cluster member, linked to facility).

### Risk perception and uncertainty

The decision to test for HIV, either in a facility or using a self-test kit, was also often driven by a perceived need to know one’s status. Some participants cited specific behaviors, such as having unprotected sex, a partner who was living with HIV, or multiple sexual partners, that prompted feelings of vulnerability to HIV infection. One participant noted that he had “refused to use condoms ever”, and after some time, decided it was time to test himself to know his HIV status (Control arm, cluster member, linked to facility). Another participant decided to test because he did not feel well: “I was just suspecting my body, so I decided to go [test] … I was also having some slight headache, so I felt that I should just go and get tested” (Control arm, cluster member, unlinked to facility).

In a few cases, HIVST provided a way to resolve uncertainties that stemmed from local rumors or misconceptions. A participant already on antiretroviral therapy (ART) described testing himself to see if the rumor he had heard was true, that HIV could become undetectable, or even result in a negative test, after long-term treatment. “There is a rumor that if you take medicine for long, the virus is usually suppressed so you can be found negative”, he explained. “I still found I was positive” (Intervention arm, cluster member, linked to facility).

### Role of promoters and peer influence

Promoters, particularly in the intervention arm, described how their training increased their own motivation to test and positioned them to influence peers. One promoter shared that after the training, he realized “it is better to know your status earlier than live without knowing your status” (Intervention arm, promoter, linked to facility). Cluster members, in turn, saw their promoters as trusted friends, rather than authority figures, who normalized testing and helped reduce fears about the process. Promoters also described encouraging one another, sometimes collectively confronting fear around HIVST use:

Respondent: The reason I had decided is because now … what gave me the most courage was my fellow promoter; he came before he tested himself as he was about to go test and he also told me that he is scared, so I told him, “Are you also as scared as I am then?” [Giggles] so I also just told him that, “We should just be courageous since we had already been told how to use the kit, if it were to kill, then those people would have told us that it kills.”

Interviewer: What was he scared of?

Respondent: He was also scared that he might use that thing and end up killing him, and I told him that, “No, if at all this thing was killing people, then the doctors would not have given US things that are Going to kill US” so, I told him that we should just try and go and check our result because those doctors are only saving our lives. (Intervention arm, promoter, linked to facility)

Experiences with HIV testing varied across study arms. Men in control clusters, who were required to attend a clinic for HIV testing and often faced barriers, such as traveling long distances to clinics, that undermined their ability to test. One control arm participant described traveling frequently for fishing across the Ugandan border, which made linking to a clinic difficult for HIV testing: “I come see my family and then go back … I haven’t taken my time to go [for HIV testing] but I am about to” (Control arm, promoter, unlinked to facility). Those who preferred HIVST were unable to access kits at health facilities due to stock-outs. These challenges highlighted the continued importance of not only making HIVST kits physically accessible but also ensuring that men feel socially supported and structurally enabled to act on their health concerns.

## Linking to HIV services: motivations and barriers

### Perceived HIV risk and prevention motivations as drivers of linkage

Following HIVST, decisions to seek confirmatory testing were influenced by perceived HIV risk, interest in prevention and social accountability. Some men described testing after a potential exposure to HIV, such as unprotected sex, or out of general concern for their health given, the realities of life in high HIV-prevalence fishing communities. One participant reflected on the perceived risks of his environment and the need for self-protection, linking this awareness directly to his decision to pursue PrEP:

When I looked at the life that we were living on this beach … the beach has no truth. […] I saw that PrEP had been brought, so I decided that I should take the PrEP … because faithfulness may not work for two people well. […] I realized that if I take it, then I will be protected.

(Intervention arm, cluster member, linked to facility)

In addition to individual risk perception, some men described relational considerations as shaping linkage decisions. Participants noted that confirmatory testing enabled discussions with romantic partners about HIV risk and prevention, including opportunities for joint testing or coordinated health decisions, situating linkage within broader relationship dynamics rather than solely individual choice.

### Social accountability to promoters and intervention-related support

For some men, transport vouchers reinforced linkage not only by reducing financial barriers but by shaping the timing of clinic attendance. Promoters described reminding cluster members that transport costs were covered and that the opportunity to use the voucher was time-limited, framing confirmatory testing as something that needed to be acted on promptly rather than deferred. In this way, vouchers functioned alongside peer encouragement and accountability to prioritize linkage amid competing work and mobility demands.

I went because of what I was told to do. These things are for the study, and you should do as told because the person who has given you instructions didn’t do it for nothing.

(Intervention arm, cluster member, linked to facility)

Some men noted that transport vouchers operated not only as financial support but also as a source of urgency, particularly for those who might otherwise postpone confirmatory testing. Promoters described actively encouraging cluster members to link by reminding them that transport costs were covered and that the opportunity to use the voucher was time-limited. These reminders framed clinic attendance as something that needed to be acted on promptly rather than deferred, reinforcing linkage as a near-term priority amid competing work and mobility demands.

### Confirmatory testing as reassurance and result verification

For others, confirmatory testing served as a means of validating the accuracy of HIVST results, particularly given concerns about potential user error. In these cases, linkage was motivated less by perceived risk and more by a desire for reassurance from a healthcare provider:

Sometimes … the self-test kit can give you the wrong results. So I said, let me go and confirm if what I did at home matches the doctor’s results. […] When I reached there, the doctor who was there also tested me again and it turned out that my self-test results and his results were the same.

(Intervention arm, promoter, linked to facility)

### Work demands, mobility and opportunity costs as persistent barriers

Despite these motivations, consistently described work schedules and frequent mobility are major barriers to linkage. Long distances to clinics, travel across fishing sites and unpredictable work hours made clinic attendance difficult. One participant explained:

The reason why I did not confirm … how to go to the other side [travel to a distant health center] was difficult. It would have been better if I got that report in this nearest health center.

(Intervention arm, cluster member, linked to facility)

Structural challenges were compounded by economic pressures. Participants frequently described prioritizing income generation over clinic visits, particularly when healthcare seeking was perceived as time-consuming or unlikely to result in immediate benefit. For many men, decisions about confirmatory testing were shaped by the opportunity costs of missing work, travel time to clinics and uncertainty about what services would be gained by attending:

Here at the beach people are too busy. […] If they are not going to get anything from where they are going, some people won’t go. […] It just depends on someone’s priorities.

(Control arm, promoter, unlinked to facility)

Control-arm participants, in particular, described delaying or foregoing confirmatory testing due to work schedules, frequent mobility, or the belief that repeated negative self-test results reduced the need for clinic-based confirmation:

“I thought it wasn’t necessary that I visit a health facility [for confirmatory testing]”, one participant noted. “Also, depending on my job, I couldn’t get the time”.

(Control arm, promoter, unlinked to facility)

Together, these accounts highlight how men weigh competing demands and assess the value of clinic-based services in context. While HIVST made initial testing more accessible, linkage to HIV care and prevention remained shaped by HIV risk perception, resource constraints and social obligations.

## Discussion

This study examined motivations and barriers to HIVST and subsequent linkage to HIV services among a highly mobile population of Kenyan fishermen. Consistent with prior work, HIVST was widely valued as a private and accessible testing option. However, linkage to confirmatory testing and HIV services following HIVST was uneven. Decisions about linkage were shaped by perceived HIV risk, interest in prevention, encouragement from intimate partners and accountability to trusted peers who served as promoters. At the same time, structural constraints, including occupational mobility, transportation challenges and competing livelihood priorities, frequently disrupted follow-up after testing.

While prior studies have documented barriers to HIVST and linkage among men in sub-Saharan Africa (Adeagbo et al., 2024; Choko et al., 2019; Hlongwa et al., 2020), few have focused specifically on highly mobile fishing populations, where fishermen balance with occupational mobility and economic precarity in their healthcare decision-making. Our findings extend this literature by demonstrating how HIVST addressed these constraints by enabling men to test privately and autonomously, outside of clinic settings that were often inaccessible due to work schedules or stigma. Building on baseline qualitative work from the Owete trial that documented anticipated acceptability of HIVST, this study provides evidence of how HIVST was taken up and acted upon in practice (Lewis-Kulzer et al., 2025). For many fishermen, self-testing represented a way to maintain some control over health decision-making despite limited engagement with facility-based services.

Linkage outcomes following HIVST have been shown to vary widely, with the strongest effects observed in interventions that combine HIVST with structural support and peer or lay-provider follow-up (Adeagbo et al., 2024; Hlongwa et al., 2023). Prior studies have shown that peer and lay provider delivery of HIVST can increase uptake, acceptability and engagement with care by leveraging trust, shared social context and reduced stigma (Ayala et al., 2021; Indravudh et al., 2021; Matovu et al., 2020). Our findings align with this evidence while offering further insight into how peer-led strategies operate in highly mobile fishing communities. Participants described promoters not merely as distributors of HIVST kits or transport vouchers, but as trusted peers whose reminders, expectations and social proximity shaped decisions about both testing and subsequent linkage. In this context, promoters functioned as embedded network actors and credible change agents who reduced uncertainty around HIVST and reinforced its legitimacy within existing social ties (Latkin & Knowlton, 2015; Valente, 2010). In this context, promoters functioned as embedded network actors and credible change agents who reduced uncertainty around HIVST and reinforced its legitimacy within existing social ties, consistent with diffusion theory emphasizing peer communication and opinion leadership in the spread of preventive innovations (Rogers, 2002). These findings suggest that linkage interventions must move beyond addressing structural barriers alone and instead account for how social accountability, trust and uncertainty shape follow-up after self-testing, particularly in settings characterized by mobility and economic precarity.

Our findings also highlight the importance of situating HIVST within a broader HIV prevention continuum that includes PrEP initiation and sustained engagement. Several participants linked confirmatory testing to interest in PrEP, framing linkage not solely as a diagnostic step but as an entry point into prevention services. This aligns with growing evidence that HIVST can function as a gateway to PrEP when confirmatory testing and prevention counseling are accessible and well-integrated (Indravudh et al., 2021; Ongolly et al., 2021; Pintye et al., 2019). However, the inconsistent linkage observed in this study suggests that without additional support, HIVST alone may be insufficient to translate prevention interest into PrEP uptake among highly mobile men. Interventions that explicitly integrate HIVST distribution with facilitated PrEP referral, peer follow-up and flexible service delivery may be particularly important for sustaining engagement in prevention among fishing populations.

Comparative evidence from HIVST linkage models in Malawi, Uganda and Zambia provides additional context for interpreting these findings. Studies in Malawi and Zambia have shown that community-based and peer-facilitated HIVST models can improve linkage when combined with active follow-up, home-based confirmatory testing, or financial incentives (Choko et al., 2019; Hayes et al., 2017; Hlongwa et al., 2020). In Uganda, network-based and peer-led HIVST approaches in fishing and mobile populations have demonstrated improved feasibility and acceptability, though linkage remains sensitive to mobility, opportunity costs and service accessibility (Matovu et al., 2025). Compared to these models, the Owete intervention underscores the added value of leveraging existing social networks not only to distribute HIVST kits, but to sustain accountability and motivation across subsequent steps in the prevention and care continuum. Together, these studies suggest that while HIVST can effectively expand testing access, durable linkage requires interventions that are both socially embedded and structurally responsive to men’s livelihoods.

Although participants primarily framed decisions about linkage in terms of work demands, time constraints and economic priorities, these accounts can be situated within broader gendered expectations around masculinity and breadwinning in fishing communities. Prior research has shown that men in similar settings often view income generation as a core marker of responsibility and adulthood, while healthcare seeking is deferred unless illness directly interferes with the ability to work (Sileo, Reed, et al., 2019a; Tumwine et al., 2020). In this context, men’s prioritization of work over confirmatory testing may reflect not only economic necessity but also gendered norms that position healthcare as secondary to fulfilling their role as providers. While participants did not explicitly describe these decisions in gendered terms, situating their narratives within this broader literature helps explain why even accessible services, such as HIVST, may not consistently translate into linkage without additional structural or social support.

Our findings suggest that transport vouchers functioned through more than a simple reduction of financial barriers. Several participants described how the time-limited nature of the vouchers created a sense of urgency that motivated timely linkage to confirmatory testing and services. This mechanism resembles deadline or time-bound incentive effects described in behavioral economics, in which individuals are more likely to act when an opportunity is perceived as temporary rather than open-ended (Ariely & Wertenbroch, 2002; Karlan & Zinman, 2009). Similar dynamics have been observed in health behavior interventions, including HIV-related service uptake, where incentives and structured timelines shape prioritization of care amid competing demands (Adeagbo et al., 2024). In this context, vouchers appeared to operate both as structural support and as a behavioral prompt that encouraged men to prioritize clinic attendance amid competing work and mobility demands. It is important to note that this urgency effect was shaped by the study context, including clear timelines for voucher use and follow-up expectations communicated by promoters. In real-world implementation, transport support may not always be time-bound or embedded within a research framework. Future HIVST linkage strategies should therefore consider whether incorporating time-limited or clearly structured support, even in the absence of monetary incentives, could help sustain linkage while remaining feasible for routine service delivery.

This study has limitations. Although qualitative data were collected at multiple time points in the parent study, the present analysis draws on interviews conducted at a single follow-up period, limiting longitudinal insight beyond self-reported reflections on behavior change over time. We did not include interviews with healthcare providers or clinic staff, which limits our understanding of how provider attitudes, clinic capacity, or health system constraints may have influenced men’s decisions to link to HIV services. In addition, no men in our sample reported receiving their first HIV-positive test result from the self-test, so we were unable to examine linkage behaviors following a new reactive result. Future research should incorporate the views of healthcare providers and include individuals who receive a reactive HIVST result to better understand the factors influencing linkage following HIVST in similar settings. Findings should also be interpreted in the context of the study’s nested design within the Owete trial, which may have influenced how participants described their experiences with HIVST and linkage to services. Participants may have emphasized socially desirable behaviors, such as testing or linkage, particularly when interviewed by researchers perceived as connected to the intervention. In addition, interpretation of findings was shaped by the analytic team’s backgrounds in HIV prevention research and prior engagement with the trial. To mitigate potential bias, we used team-based coding, reflexive discussion during analysis and attention to divergent or contradictory accounts. Nonetheless, researchers’ assumptions and relationships with the study context may have shaped which themes received greater analytic attention.

## Conclusions

Our study found that HIVST was highly acceptable among fishermen and that social network–based, peer-led delivery motivated uptake and linkage to HIV services. Trained “promoters” played a central role in motivating testing and linkage by distributing HIVST kits and transport vouchers and by framing testing as a responsible, community-supported act. Their involvement fostered trust and accountability, with many men linking to care out of respect for the promoters who had encouraged them. For fishermen, barriers to timely linkage to services following HIVST included long distances to clinics, frequent mobility for work and gender norms prioritizing work over health. Future research should explore how social network interventions can be harnessed to promote sustained engagement across the HIV prevention and treatment cascade.

## Figures and Tables

**Table 1.** Characteristics of Owete qualitative participants at 3-month follow-up, *N* = 64.

Characteristic	*N*(%)
Age, years (median, IQR)	34 (29.0–39.1)
Beach Community	
Beach 1	18 (28.6)
Beach 2	23 (36.5)
Beach 3	22 (34.9)
Marital Status	
Married	55 (85.9)
Unmarried but partnered	1 (1.6)
Single/divorced/widowed	8 (12.5)
Education	
Primary or less	45 (70.3)
Some/all secondary	15 (23.4)
Secondary or higher	4 (6.3)
Polygynous marriage	13 (23.6)
Wealth quintiles	
Lowest quintile	12 (20.0)
All other	48 (80.0)
Sexual risk behaviour past 6 months	
Any concurrent sexual partner	24 (38.7)
Any high risk sexual partner	8 (12.7)
No condom use with any partner	36 (57.14)
Ever tested for HIV	60 (93.8)
Study arm	
Intervention	31 (48.4)
Control	33 (51.6)

## Data Availability

De-identified study data and a data dictionary will be made available in a secure online repository approximately 1 year after completion of the ongoing trial (NCT04772469) following Owete Scientific Committee approval of a concept sheet summarizing the analyses to be done, with a signed data access agreement. Further inquiries can be directed to the Owete Scientific Committee via Carol. Camlin@ucsf.edu.

## References

[R1] Adeagbo OA, Badru OA, Nkfusai CN, & Bain LE (2024). Effectiveness of linkage to care and prevention interventions following HIV self-testing: A global systematic review and meta-analysis. AIDS and Behavior, 28(4), 1314–1326. 10.1007/s10461-023-04162-5 37668817

[R2] Ariely D, & Wertenbroch K (2002). Procrastination, deadlines, and performance: Self-control by precommitment. Psychological Science, 13(3), 219–224. 10.1111/1467-9280.00441 12009041

[R3] Ayala G, Sprague L, van der Merwe LL-A, Thomas RM, Chang J, Arreola S, Davis SLM, Taslim A, Mienies K, Nilo A, Mworeko L, Hikuam F, de Leon Moreno CG, & Izazola-Licea JA (2021). Peer- and community-led responses to HIV: A scoping review. PLoS ONE, 16(12), e0260555. 10.1371/journal.pone.0260555 34852001 PMC8635382

[R4] Camlin CS, Sheira LA, Kwena ZA, Charlebois ED, Agot K, Moody J, Ayieko B, Gutin SA, Ochung A, Olugo P, Lewis-Kulzer J, Nishimura H, Gandhi M, Bukusi EA, & Thirumurthy H (2025). The effect of a social network-based intervention to promote HIV testing and linkage to HIV services among fishermen in Kenya: A cluster-randomised trial. Lancet Global Health, 13(4), e669–e678. 10.1016/s2214-109x(24)00539-4 40155104 PMC12431777

[R5] Chipungu J, Bosomprah S, Zanolini A, Thimurthy H, Chilengi R, Sharma A, & Holmes CB (2017). Understanding linkage to care with HIV self-test approach in Lusaka, Zambia – a mixed method approach. PLoS ONE, 12(11), e0187998. 10.1371/journal.pone.0187998 29149194 PMC5693414

[R6] Choko AT, Corbett EL, Stallard N, Maheswaran H, Lepine A, Johnson CC, Sakala D, Kalua T, Kumwenda M, Hayes R, & Fielding K (2019). HIV self-testing alone or with additional interventions, including financial incentives, and linkage to care or prevention among male partners of antenatal care clinic attendees in Malawi: An adaptive multi-arm, multi-stage cluster randomised trial. PLoS Medicine, 16(1), e1002719. 10.1371/journal.pmed.1002719 30601823 PMC6314606

[R7] Conserve DF, Muessig KE, Maboko LL, Shirima S, Kilonzo MN, Maman S, & Kajula L (2018). Mate Yako Afya Yako: Formative research to develop the Tanzania HIV self-testing education and promotion (Tanzania STEP) project for men. PLoS ONE, 13(8), e0202521. 10.1371/journal.pone.0202521 30148846 PMC6110473

[R8] d’Elbée M, Indravudh PP, Mwenge L, Kumwenda MM, Simwinga M, Choko AT, Hensen B, Neuman M, Ong JJ, Sibanda EL, Johnson CC, Hatzold K, Cowan FM, Ayles H, Corbett EL, & Terris-Prestholt F (2018). Preferences for linkage to HIV care services following a reactive self-test: Discrete choice experiments in Malawi and Zambia. AIDS, 32(14), 2043–2049. 10.1097/qad.0000000000001918 29894386 PMC7610994

[R9] Gale NK, Heath G, Cameron E, Rashid S, & Redwood S (2013). Using the framework method for the analysis of qualitative data in multi-disciplinary health research. BMC Medical Research Methodology, 13(1), 117. 10.1186/1471-2288-13-117 24047204 PMC3848812

[R10] Hayes R, Floyd S, Schaap A, Shanaube K, Bock P, Sabapathy K, Griffith S, Donnell D, Piwowar-Manning E, El-Sadr W, Beyers N, Ayles H, & Fidler S (2017). A universal testing and treatment intervention to improve HIV control: One-year results from intervention communities in Zambia in the HPTN 071 (PopART) cluster-randomised trial. PLoS Med, 14(5), e1002292. 10.1371/journal.pmed.1002292 28464041 PMC5412988

[R11] Hlongwa M, Hlongwana K, Makhunga S, Choko AT, Dzinamarira T, Conserve D, & Tsai AC (2023). Linkage to HIV care following HIV self-testing among men: Systematic review of quantitative and qualitative studies from six countries in Sub-Saharan Africa. AIDS and Behavior, 27(2), 651–666. 10.1007/s10461-022-03800-8 36094641 PMC9466308

[R12] Hlongwa M, Mashamba-Thompson T, Makhunga S, & Hlongwana K (2020). Barriers to HIV testing uptake among men in Sub-Saharan Africa: A scoping review. African Journal of AIDS Research, 19(1), 13–23. 10.2989/16085906.2020.1725071 32174231

[R13] Indravudh PP, Fielding K, Kumwenda MK, Nzawa R, Chilongosi R, Desmond N, Nyirenda R, Neuman M, Johnson CC, Baggaley R, Hatzold K, Terris-Prestholt F, & Corbett EL (2021). Effect of community-led delivery of HIV self-testing on HIV testing and antiretroviral therapy initiation in Malawi: A cluster-randomised trial. PLoS medicine, 18(5), e1003608. 10.1371/journal.pmed.1003608 33974621 PMC8112698

[R14] Johnson C, Baggaley R, Forsythe S, van Rooyen H, Ford N, Napierala Mavedzenge S, Corbett E, Natarajan P, & Taegtmeyer M (2014). Realizing the potential for HIV self-testing. AIDS and Behavior, 18(S4), 391–395. 10.1007/s10461-014-0832-x 24986599

[R15] Johnson CC, Kennedy C, Fonner V, Siegfried N, Figueroa C, Dalal S, Sands A, & Baggaley R (2017). Examining the effects of HIV self-testing compared to standard HIV testing services: A systematic review and meta-analysis. Journal of the International AIDS Society, 20(1), 21594. 10.7448/ias.20.1.21594 28530049 PMC5515051

[R16] Karlan D, & Zinman J (2009). Observing unobservables: Identifying information asymmetries with a consumer credit field experiment. Econometrica, 77(6), 1993–2008. 10.3982/ecta5781

[R17] Kenya Ministry of Health. (2020). Kenya HIV estimates report 2020. Kenya Ministry of Health.

[R18] Kenya Ministry of Health. (2022). Kenya population-based HIV impact assessment (KENPHIA) 2018: Finalreport.

[R19] Korte JE, Kisa R, Vrana-Diaz CJ, Malek AM, Buregyeya E, Matovu JK, Kagaayi J, Musoke W, Chemusto H, Mukama SC, Ndyanabo A, Mugerwa S, & Wanyenze RK (2020). HIV oral self-testing for male partners of women attending antenatal care in central Uganda: Uptake of testing and linkage to care in a randomized trial. JAIDS Journal of Acquired Immune Deficiency Syndromes, 84(3), 271–279. 10.1097/qai.0000000000002341 32168168

[R20] Kwena ZA, Camlin CS, Shisanya CA, Mwanzo I, & Bukusi EA (2013). Short-term mobility and the risk of HIV infection among married couples in the fishing communities along Lake Victoria, Kenya. PLoS ONE, 8(1), e54523. 10.1371/journal.pone.0054523 23336005 PMC3545885

[R21] Latkin CA, & Knowlton AR (2015). Social network assessments and interventions for health behavior change: A critical review. Behavioral Medicine, 41(3), 90–97. 10.1080/08964289.2015.1034645 26332926 PMC4786366

[R22] Lewis-Kulzer J, Olugo P, Gutin SA, Kwena ZA, Nishimura H, Thorp M, Agot K, Ayieko B, Bukusi EA, Oluoch L, Angawa D, Thirumurthy H, & Camlin CS (2025). “There is no need to leave the beach to test”: A qualitative study of HIV self-testing knowledge and acceptability of HIV self-test kit distribution among social networks of fishermen in Western Kenya. BMC Public Health, 25(1), 1005. 10.1186/s12889-025-22136-1 40087617 PMC11908045

[R23] Matovu JK, Nambuusi A, Nakabirye S, Wanyenze RK, & Serwadda D (2020). Formative research to inform the development of a peer-led HIV self-testing intervention to improve HIV testing uptake and linkage to HIV care among adolescents, young people and adult men in Kasensero fishing community, Rakai, Uganda: A qualitative study. BMC Public Health, 20(1), 1582. 10.1186/s12889-020-09714-1 33081735 PMC7576713

[R24] Matovu JKB, Namwama AT, Kemigisha L, Taasi G, Nakabugo J, Wandabwa J, Bogart LM, Fakier N, Wanyenze RK, Olupot-Olupot P, Musinguzi J, & Serwadda D (2025). Feasibility, acceptability and preliminary effects of a social network-based, peer-led HIV self-testing intervention among men in two Ugandan fishing communities, 2022. Arch Public Health, 83(1), 23. 10.1186/s13690-025-01511-9 39849572 PMC11761786

[R25] Olugo P, Nishimura H, Thorp M, Lewis-Kulzer J, Ayieko B, Agot K, Kwena ZA, Camlin CS, & Gutin SA (2025). Misconceptions and limited experience with HIV pre-exposure prophylaxis (PrEP) among fishermen in Western Kenya: A qualitative study. AIDS Care, 37(5), 1–10. 10.1080/09540121.2025.2480149 40131995 PMC12479176

[R26] Ongolly FK, Dolla A, Ngure K, Irungu EM, Odoyo J, Wamoni E, Peebles K, Mugwanya K, Mugo NR, Bukusi EA, Morton J, Baeten JM, & O’Malley G (2021). “I just decided to stop:” understanding PrEP discontinuation among individuals initiating PrEP in HIV care centers in Kenya. Journal of Acquired Immune Deficiency Syndromes, 87(1), e150–e158. 10.1097/qai.0000000000002625 33492024 PMC8026512

[R27] Pintye J, Drake AL, Begnel E, Kinuthia J, Abuna F, Lagat H, Dettinger J, Wagner AD, Thirumurthy H, Mugwanya K, Baeten JM, & John-Stewart G (2019). Acceptability and outcomes of distributing HIV self-tests for male partner testing in Kenyan maternal and child health and family planning clinics. AIDS, 33(8), 1369–1378. 10.1097/qad.0000000000002211 30932954 PMC6546533

[R28] Rogers EM (2002). Diffusion of preventive innovations. Addictive Behaviors, 27(6), 989–993. 10.1016/s0306-4603(02)00300-3 12369480

[R29] Rujumba J, Homsy J, Mbazzi FB, Namukwaya Z, Amone A, Rukundo G, Katabira E, Byamugisha J, Fowler MG, & King RL (2021). Pregnant women, their male partners and health care providers’ perceptions of HIV self-testing in Kampala, Uganda: Implications for integration in prevention of mother-to-child transmission programs and scale-up. PLoS ONE, 16(6), e0253616. 10.1371/journal.pone.0253616 34185799 PMC8241041

[R30] Saunders B, Sim J, Kingstone T, Baker S, Waterfield J, Bartlam B, Burroughs H, & Jinks C (2018). Saturation in qualitative research: Exploring its conceptualization and operationalization. Quality & Quantity, 52(4), 1893–1907. 10.1007/s11135-017-0574-8 29937585 PMC5993836

[R31] Sheira LA, Kwena ZA, Charlebois ED, Agot K, Ayieko B, Gandhi M, Bukusi EA, Thirumurthy H, & Camlin CS (2022). Testing a social network approach to promote HIV self-testing and linkage to care among fishermen at Lake Victoria: Study protocol for the Owete cluster randomized controlled trial. Trials, 23(1), 463. 10.1186/s13063-022-06409-3 35668499 PMC9169331

[R32] Sileo KM, Reed E, Kizito W, Wagman JA, Stockman JK, Wanyenze RK, Chemusto H, Musoke W, Mukasa B, & Kiene SM (2019a). Masculinity and engagement in HIV care among male fisherfolk on HIV treatment in Uganda. Culture, Health & Sexuality, 21(7), 774–788. 10.1080/13691058.2018.1516299 PMC6513725 30422078

[R33] Sileo KM, Wanyenze RK, Kizito W, Reed E, Brodine SK, Chemusto H, Musoke W, Mukasa B, & Kiene SM (2019b). Multi-level determinants of clinic attendance and antiretroviral treatment adherence among fishermen living with HIV/AIDS in communities on Lake Victoria, Uganda. AIDS and Behavior, 23(2), 406–417. 10.1007/s10461-018-2207-1 29959718 PMC6492274

[R34] Sileo KM, Wanyenze RK, Mukasa B, Musoke W, & Kiene SM (2021). The intersection of inequitable gender norm endorsement and HIV stigma: Implications for HIV care engagement for men in Ugandan fishing communities. AIDS and Behavior, 25(9), 2863–2874. 10.1007/s10461-021-03176-1 33566214 PMC8353000

[R35] Silva JM (2013). Coming up short: Working-class adulthood in an age of uncertainty. Oxford University Press.

[R36] Socio Cultural Research Consultants. (2023). Dedoose version 9.0.107, cloud application for managing, analyzing, and presenting qualitative and mixed method research data. www.dedoose.com

[R37] Tumwine C, Aggleton P, & Bell S (2020). Enhancing HIV prevention: Social support, access to, and use of HIV testing, treatment, and care services in fishing communities around Lake Victoria, Uganda. AIDS Education and Prevention, 32(3), 196–211. 10.1521/aeap.2020.32.3.196 32749878

[R38] Valente TW (2010). Social networks and health: Models, methods, and applications. Oxford University Press.

